# *Bordetella avium*-associated endophthalmitis: case report

**DOI:** 10.1186/s12879-021-06546-1

**Published:** 2021-08-19

**Authors:** Rui Zhang, Liping Hu, Chong Xu, Jianhua Wu, Changzhong Xu, Chao Feng

**Affiliations:** Aier Eye Hospital of Wuhan University, Wuhan, Hubei China

**Keywords:** *Bordetella avium*, Endophthalmitis, Pars plana vitrectomy, Nanopore targeted sequencing, Case report

## Abstract

**Background:**

*Bordetella avium*, an aerobic bacterium that rarely causes infection in humans, is a species of *Bordetella* that generally inhabits the respiratory tracts of turkeys and other birds. It causes a highly contagious bordetellosis. Few reports describe *B. avium* as a causative agent of eye-related infections.

**Case presentation:**

We report a case of acute infectious endophthalmitis associated with infection by *B. avium* after open trauma. After emergency vitrectomy and subsequent broad-spectrum antibiotic treatment, the infection was controlled successfully, and the patient’s vision improved.

**Conclusions:**

*B. avium* can cause infection in the human eye, which can manifest as acute purulent endophthalmitis. Nanopore targeted sequencing technology can quickly identify this organism. Emergency vitrectomy combined with lens removal and silicone oil tamponade and the early application of broad-spectrum antibiotics are key for successful treatment.

## Background

*Bordetella avium* is an aerobic bacterium that rarely causes infection in humans, although it has been isolated from respiratory specimens from patients [1, 2]. *B. avium* is member of the *Bordetella* genus. The first three species to be described (*B. pertussis*, *B. parapertussis*, *B. bronchiseptica*) are sometimes referred to as ‘classical species' [3]. *B. avium* has a global distribution and mainly affects young domesticated turkeys [4]. *B. avium*-associated disease in the human eye has not yet been reported.

Here, we present a case of acute infectious endophthalmitis caused by infection with *B. avium* after open trauma. The pathogen was confirmed by nanopore targeted sequencing.

## Case presentation

**Fig. 1:**
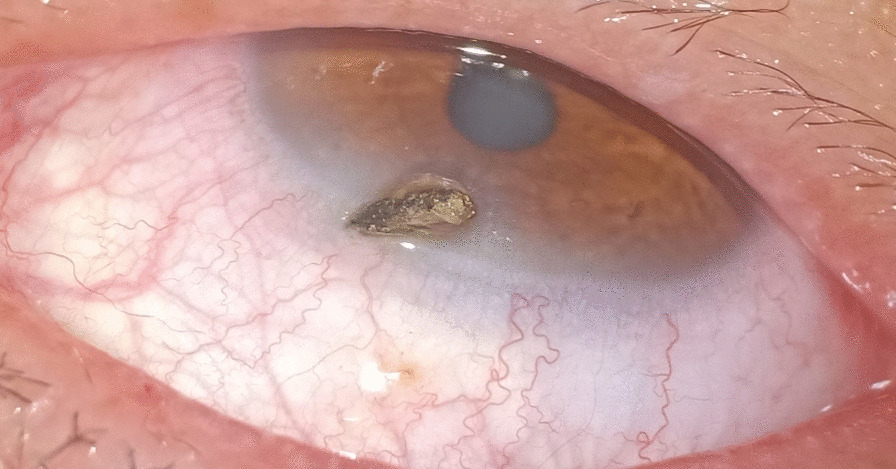
The foreign body was located near the upper limbus and penetrated the whole cornea

**Fig. 2:**
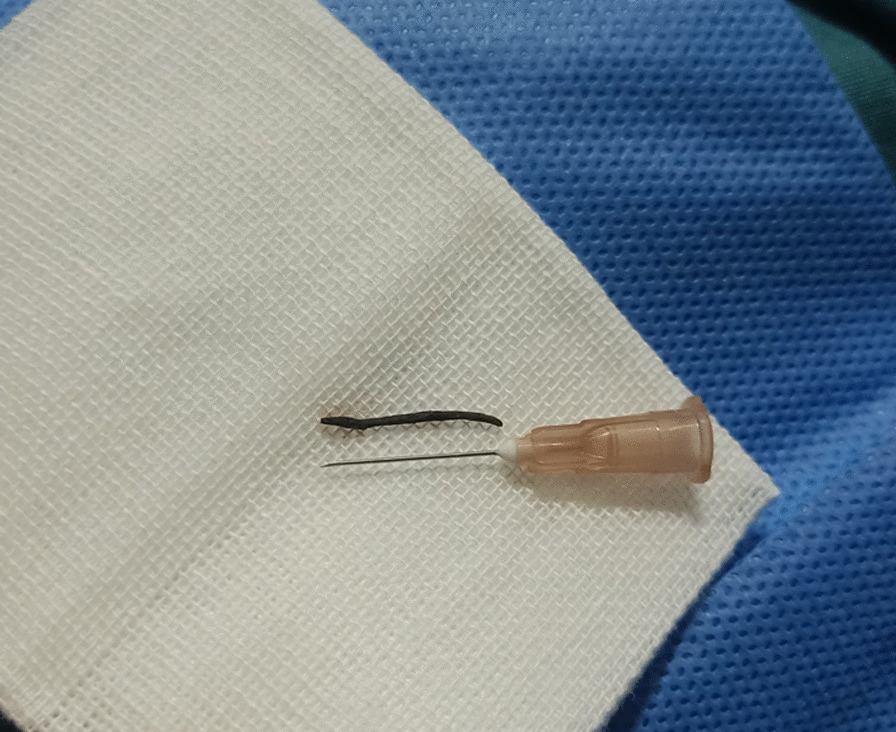
The patient underwent foreign body removal with primary debridement and suturing

A 47-year-old man was admitted to the Department of Vitreous & Retina in Aier Eye Hospital of Wuhan University, for impaired vision and eye pain in his right eye for 2 days. Three days before admission, he received a penetrating injury to the right eye and underwent an emergency operation for foreign body removal combined with debridement and suturing in a local hospital (Figs. 1 and 2). He had previously suffered from acute hepatitis that had been cured and had a history of vocal cord polypectomy and no chronic diseases, such as hypertension or diabetes mellitus. A complete review of symptoms, including chills, fevers, weight loss, and night sweats, and a history of contacts with ill persons were unremarkable.

**Fig. 3:**
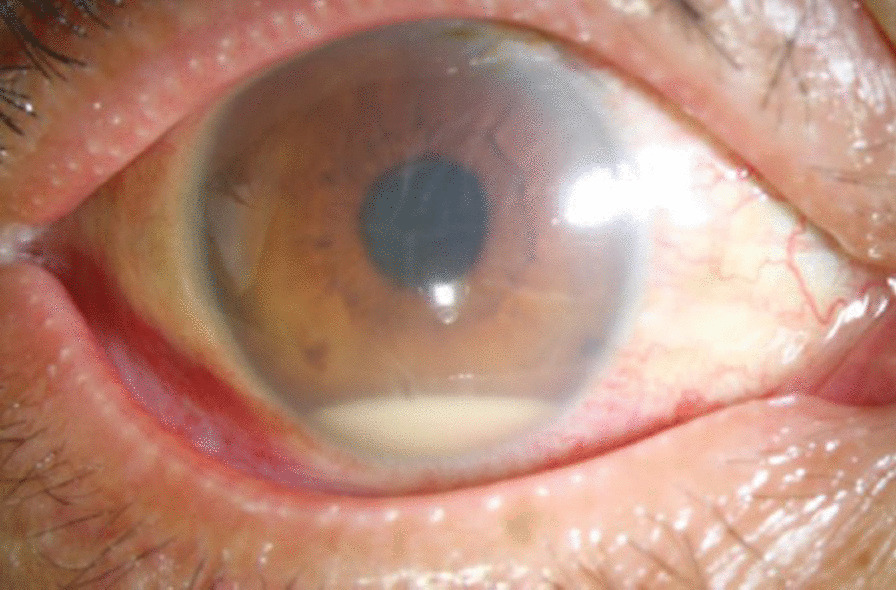
The patient had conjunctival hyperemia, corneal edema and hypopyon on admission to our department

**Fig. 4:**
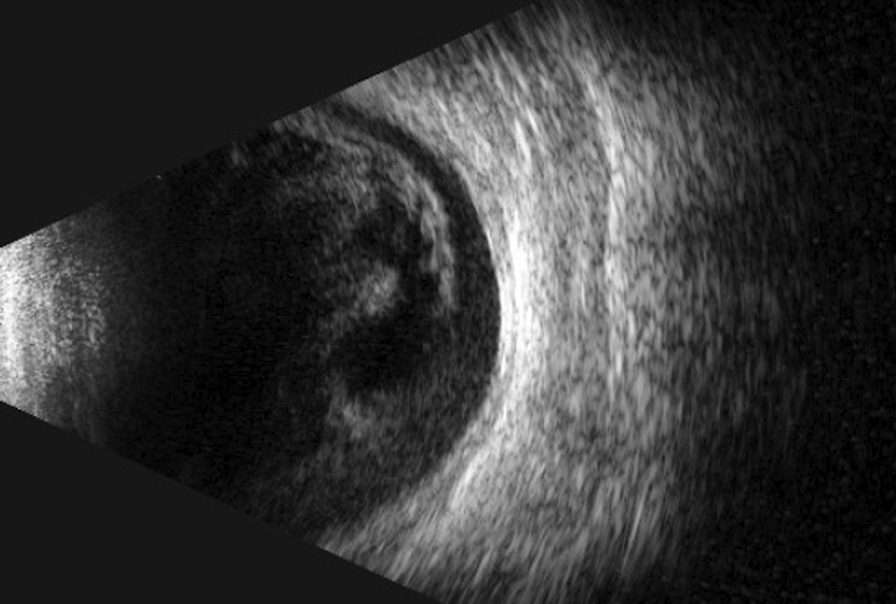
B-scan ultrasonography showed extensive vitreous opacities and an attached retina

Visual acuity in the right eye was hand motion at 30 cm, and in the left eye was 20/20. Intraocular pressure was normal bilaterally. In the right eye, an anterior segment examination showed mild conjunctival hyperemia, corneal edema, a hypopyon and an exudative membrane on the anterior surface of the crystalline lens (Fig. 3). A posterior segment examination could not be performed due to the opacity of the refractive medium. B-scan ultrasonography showed extensive vitreous opacities and an attached retina (Fig. 4).

The results of the complete blood count showed that the white blood cell count was normal, the neutrophil count was normal, and the neutrophil ratio was increased, while the lymphocyte count, monocyte count, eosinophil count and their respective ratios were all decreased.

The patient was diagnosed with acute infectious endophthalmitis and underwent pars plana vitrectomy combined with lens removal and silicone oil tamponade in the right eye. Vancomycin was added to the balanced salt solution. A vitreous sample was sent for isolation-culture and nanopore targeted sequencing (Wuhan Dgensee Clinical Laboratory Co., Ltd.).

Using biochemical identification method, culture analysis showed gram-negative bacilli but did not indicate the species. The antimicrobial susceptibility test showed that the minimum inhibitory concentrations (MICs) of imipenem, ceftazidime, meropenem, polymyxin B and trimethoprim/sulfa indicated susceptibility. Nanopore targeted sequencing yielded positive results for *Bordetella avium.* (This Targeted Locus Study project has been deposited at DDBJ/ENA/GenBank under the accession KETD00000000. The version described in this paper is the first version, KETD01000000.) The bacterium was additionally verified as *B. avium* by the first-generation gene sequencing method.

**Fig. 5:**
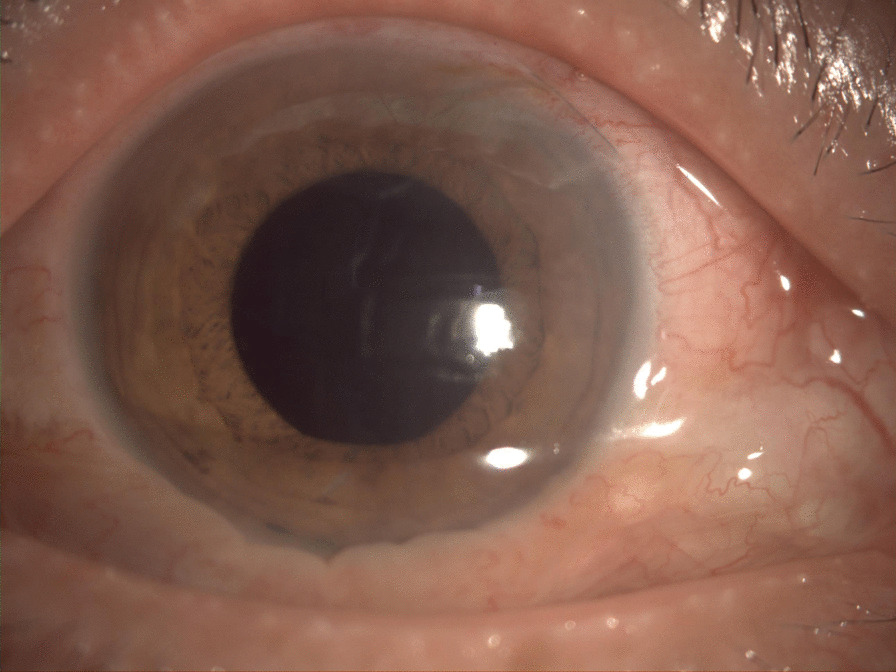
The patient had a mildly congestive and edematous bulbar conjunctiva, almost transparent cornea, and clear anterior chamber when he was discharged

**Fig. 6:**
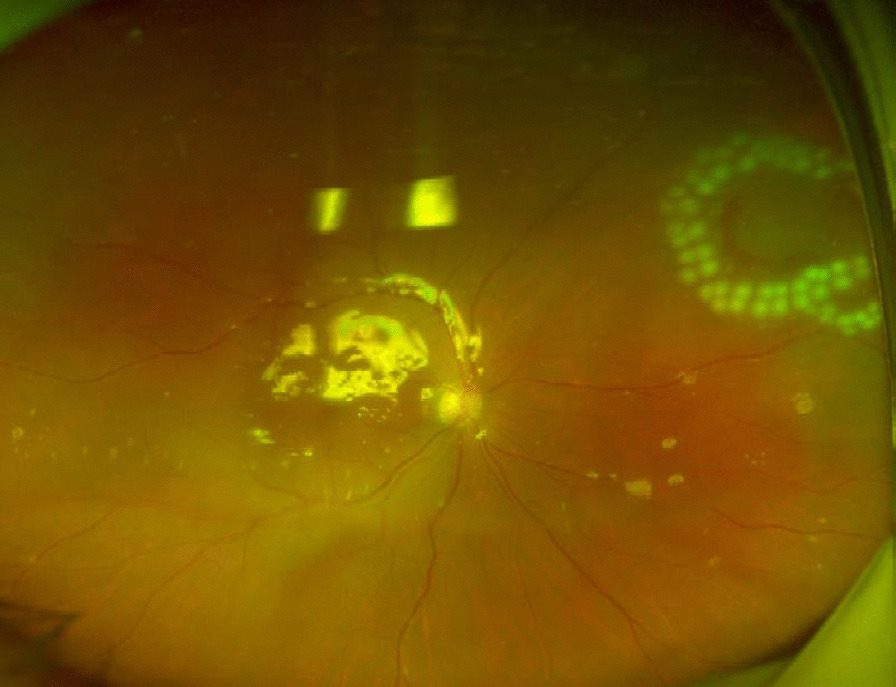
The patient had almost no obvious abnormalities in the fundus when he was discharged

The postoperative treatment was ceftazidime 1000 mg intravenously *tid*, and 0.5 % levofloxacin eye drops topical use *qid*. On the first day after the operation, we observed that the intraocular infection was effectively controlled, and the eye condition continued to improve in the following days, so topical steroidal anti-inflammatory drugs were added. The patient was discharged from the hospital one week post operation after observation of a mildly congestive and edematous bulbar conjunctiva, an almost transparent cornea, a clear anterior chamber, and a relatively healthy retina (Figs. 5 and 6). In addition, the best corrected visual acuity was 20/50.

## Discussion and conclusions

*B. avium* is a gram-negative, rod-shaped, nonfermentative, strictly aerobic, motile bacterium from the genus *Bordetella* that causes turkey coryza, an acute respiratory tract disease in young turkeys [5]. Human infections are rare, although a few case reports of respiratory infections have been published [1, 2]. No susceptibility guidelines for *Bordetella* species have been established, as there is conflicting evidence regarding susceptibility [6–9]. The optimal antimicrobial drug treatment for infection with *B. avium* is unknown, and empiric broad-spectrum antibiotic therapy might be reasonable, especially in the absence of microbiological data [10]. But ceftazidime was lacking at that time, so only vancomycin was used in the emergency surgery. The postoperative use of local antibiotics could form an effective therapeutic concentration in the anterior chamber. And the blood-retinal barrier function of the patient was temporarily damaged after trauma and surgery, the use of systemic antibiotics could allow the drug to reach the retina and choroid. These could further inhibit the reproduction of remaining bacteria in the eyeball and kill them.

According to the literature search, there are no reports of *B. avium*-related ocular infections. By obtaining the patient’s medical history, we learned that he was on a chicken farm environment at the time of injury, which is logically consistent with the detection result. We speculate that his eye was infected by contact with an object contaminated by *B. avium*. In the process of bacterial culture, the strain is not pure and the bacterial age and concentration of the bacteria to be tested are inappropriate, may be the reason that the organism couldn’t be culture identified.

Nanopore targeted sequencing, as the most recent generation of sequencing technology, sequences nucleotides in DNA or RNA by measuring the fluctuation of the current as the molecule passes through the nanopore without first amplifying the sample. It can perform one-time sequencing of a whole of a nucleic acid strand, sometimes longer than a million bases, eliminating the need for the use of short segments of only a few hundred bases. It also has the advantage of being able to read sequences directly from biological samples in real time [11, 12]. This sequencing technology is widely used for the rapid identification of viral pathogens [13], rapid gene mutation detection in those with certain diseases [14] and other applications. Previously, *B. avium* was identified mainly by isolation and culture, biochemical tests and PCR assays [1, 2, 15–17]. This was the first time that *B. avium* was identified by nanopore targeted sequencing.

We report a case of acute infectious endophthalmitis caused by infection with *B. avium*. Molecular methods enabled the successful identification of this organism, which is generally considered to have relatively low virulence; accordingly, there are no susceptibility guidelines. A combined approach of pars plana vitrectomy plus lens removal and silicone oil tamponade and empiric broad-spectrum antibiotic therapy led to a successful outcome.

## Data Availability

The datasets generated and/or analysed during the current study are available in the GenBank repository, https://www.ncbi.nlm.nih.gov/nuccore/KETD00000000.

## Abbreviations

MIC: Minimal inhibitory concentration
